# Analysis of the Implementation, User Perspectives, and Feedback From a Mobile Health Intervention for Individuals Living With Hypertension (DREAM-GLOBAL): Mixed Methods Study

**DOI:** 10.2196/12639

**Published:** 2019-12-09

**Authors:** Jordan Barsky, Rebekah Hunter, Colin McAllister, Karen Yeates, Norm Campbell, Peter Liu, Nancy Perkins, Diane Hua-Stewart, Marion A Maar, Sheldon W Tobe

**Affiliations:** 1 Department of Medicine Sunnybrook Health Sciences Centre, Sunnybrook Research Institute University of Toronto Toronto, ON Canada; 2 Perspect Management Consulting Regina, SK Canada; 3 Department of Medicine Queens University Kingston, ON Canada; 4 Department of Medicine University of Calgary Calgary, AB Canada; 5 University of Ottawa Heart Institute Ottawa, ON Canada; 6 Faculty of Medicine Northern Ontario School of Medicine Sudbury, ON Canada

**Keywords:** blood pressure, hypertension, mHealth, population groups, short message service

## Abstract

**Background:**

DREAM-GLOBAL (Diagnosing hypertension—Engaging Action and Management in Getting Lower Blood Pressure in Indigenous and low- and middle-income countries) studied a SMS text messaging–based system for blood pressure measurement and hypertension management in Canadian Aboriginal and Tanzanian communities. The use of SMS text messages is an emerging point of interest in global health care initiatives because of their scalability, customizability, transferability, and cost-effectiveness.

**Objective:**

The study aim was to assess the effect on the difference in blood pressure reduction of active hypertension management messages or passive health behavior messages. The system was designed to be implemented in remote areas with wireless availability. This study described the implementation and evaluation of technical components, including quantitative data from the transmission of blood pressure measurements and qualitative data collected on the operational aspects of the system from participants, health care providers, and community leadership.

**Methods:**

The study was implemented in six remote Indigenous Canadian and two rural Tanzanian communities. Blood pressure readings were taken by a community health worker and transmitted to a mobile phone via Bluetooth, then by wireless to a programmed central server. From the server, the readings were sent to the participant’s own phone as well. Participants also received biweekly tailored SMS text messages on their phones. Quantitative data on blood pressure reading transmissions were collected from the study central server. Qualitative data were collected by surveys, focus groups, and key informant interviews of participants, health care providers, and health leadership.

**Results:**

In Canada, between February 2014 and February 2017, 2818 blood pressure readings from 243 patients were transmitted to the central server. In Tanzania, between October 2014 and August 2015, 1165 readings from 130 patients were transmitted to the central server. The use of Bluetooth technology enabled the secure, reliable transmission of information from participants to their health care provider. The timing and frequency were satisfactory to 137 of 187 (73.2%) of participants, supporting the process of sending weekly messages twice on Mondays and Thursdays at 11 am. A total of 97.0% (164/169) of the participants surveyed said they would recommend participation in the DREAM-GLOBAL program to a friend or relative with hypertension.

**Conclusions:**

In remote communities, the DREAM-GLOBAL study helped local health care providers deliver a blood pressure management program that enabled patients and community workers to feel connected. The technical components of the study were implemented as planned, and patients felt supported in their management through the SMS text messaging and mobile health program. Technological issues were solved with troubleshooting. Overall, the technical aspects of this research program enhanced clinical care and study evaluation and were well received by participants, health care workers, and community leadership.

**Trial Registration:**

Clinicaltrials.gov NCT02111226; https://clinicaltrials.gov/ct2/show/NCT02111226.

## Introduction

### Background

There are over 1 billion individuals living with hypertension around the world, of which two-third are in developing countries [1]. Achieving the rates of hypertension control in low- and middle-income countries (LMICs), and in remote areas and vulnerable populations equal to those provided in high-income countries, remains a treatment gap that has not yet been bridged. The Global Alliance for Chronic Diseases funded and facilitated global collaborations in hypertension implementation research, and it sponsored this research, which was focused on improving the management of hypertension in LMICs and Canada’s Indigenous communities [2].

The prevalence of cardiovascular disease, obesity, diabetes, and other related conditions is significantly higher in Indigenous populations in Canada than the rest of the population. Although the prevalence of hypertension is slightly lower, rates of hypertension awareness, treatment, and control are also lower [3]. This is because of the social determinants of health, including physical inactivity, smoking, and diets that are high in sodium and low in fresh fruits/vegetables, and those specific to Indigenous peoples; this also includes poverty and the impact of colonization and the Residential School system, creating barriers to accessing health care, and an increase in behavioral risk factors for cardiovascular disease [4,5]. The ubiquity of mobile phones and mobile health presents an opportunity to overcome some of these barriers.

The use of SMS text messaging in health care interventions is becoming a point of interest because of its scalability and capacity for adaptation to various populations at a low cost [6]. Studies are finding that they are acceptable to recipients and beneficial in the management of chronic disease, but outcomes remain mixed [5,7].

### Objectives

The DREAM-GLOBAL, Diagnosing hypertension—Engaging Action and Management in Getting Lower Blood Pressure in Indigenous and low- and middle-income countries, research study was a randomized controlled trial comparing only the blood pressure lowering effect of passive health behavior messages with both active blood pressure management messages and passive health behavior messages [8]. The active messages were specific to the control or lack of control of the participant’s blood pressure. To keep the patient’s primary health care provider in the loop, the central server also sent faxes with the blood pressure readings to their office. This paper provides a technical review of the DREAM-GLOBAL intervention, discussing the development, implementation, and feedback of the system on the basis of responses from study participants, health care providers, and community health workers.

## Methods

### Design and SMS Text Message Development

The DREAM-GLOBAL study randomized participants to either active or passive SMS text messages, and the protocol has been previously published [8]. The study took place in 6 Canadian First Nations communities and 2 rural communities in Tanzania. The SMS text messages used in the study were developed from Canadian hypertension clinical practice guidelines and modified to be culturally safe and appropriate for the target population. This was carried out both in Canadian First Nations communities and in rural communities in Tanzania. All communities engaging in the study were invited to provide input on the messages to ensure that the language used was culturally appropriate, and cultural factors were taken into consideration when designing the intervention [9,10]; these factors included social, political, and historical aspects to better understand cultural differences and how these factors can affect health inequities in these rural areas of interest. An empirical decision on the frequency of the messages was made to send them twice weekly on Mondays and Thursdays at 11 am, which also helped to avoid holidays. A central server was programmed to send the messages to participants after registering with the program. The messages were not altered once the study began. The registration process was conducted by local community health workers in each community and participants recruited through hypertension screening days. The individual blood pressure measurements were taken using an automated blood pressure device with Bluetooth transmission capability. Mobile apps were developed for BlackBerry and Android wireless phones to receive the blood pressure measurements and transmit them to the central server. Upon registration into the system, the central server randomized patients to receive either passive or active SMS text messages. For a diagram of the circle of care model and the technological components, see Multimedia Appendix 1.

### Participants

The details of how the communities were identified and assessed for research readiness have been previously described [9]. The population size of the individual First Nations communities ranged from 500 to 4000. The communities were self-governed; they oversaw their own health care, provided partly by provincial and federal health ministries. Some of the remote First Nations communities had only obtained cell towers as recently as 2 years before the study; therefore, the prevalence of mobile phone use at the time was low in these communities. Participants were community members aged 18 years or older, with uncontrolled hypertension and on or off medications. They had to either have a mobile phone capable of receiving SMS text messages or be willing to carry and learn to use a basic flip phone for the study duration. Participants had to have a current primary health care provider. Blood pressure was measured with the A&D UA-767PBT-C monitor (A&D Medical), paired with a BlackBerry or an Android mobile phone app, to identify listed participants and facilitate the wireless transfer of the blood pressure to the central server.

### Ethics and Safety

Cellular safety information was outlined to the participants before the study, including adherence to a ban on handheld devices while driving. Participants were advised not to share their phone with others if they did not want anyone to know about their participation in the study, and they were also advised not to send important personal information (ie, bank account or credit card number).

Ethics approval was obtained from the Sunnybrook Health Sciences Centre Research Ethics Board (Approval Number: 953-2013) and from participating University and community ethics boards. The study was carried out according to the principles of Good Clinical Practice, the Declaration of Helsinki, and the TriCouncil Policy Statement on ethical conduct for research involving humans [11-13]. The study adhered to the principles of Ownership, Control, Access, and Possession [14]. The technology and movement of data adhered to the principles of the Personal Information Protection and Electronic Documents Act.

### Training on Use of Technology

Before the training and study launch, usability testing was conducted within the study investigator’s clinics to confirm reliability of the technology to record and transmit the blood pressure readings to the central server (see Multimedia Appendix 2 for training slides). A goal of the DREAM-GLOBAL technology was to develop a mobile phone app for a trained community health worker or community health nurse to link the mobile phone to the blood pressure measurement device and allow transmission of the reading to the central server. One blood pressure measurement device could therefore be used for multiple participants. Participants only needed an SMS text messaging–capable mobile phone. One community health worker could therefore manage many participants who did not require training on blood pressure measurement. The community health worker and home and community care nurses, after a 2-day training session, were able to recruit, consent, register participants, and take the blood pressure readings, according to guidelines, with the automated oscillometric blood pressure device [15]. The components of this training program are found in Multimedia Appendix 2. The primary outcome of the study was the difference in systolic and diastolic blood pressure from the baseline period to the last 2 months of measurement between randomized groups.

Training to carry out the study was conducted in each community by study team members at planned education sessions. Training community health workers in the study procedures included information on using the Bluetooth-enabled blood pressure machines, enrolling patients into the program, teaching patients how to use their cellular device, and communicating with their patients. They were also provided with an instructional video on how to use the mobile app and blood pressure device to take the blood pressure readings and transmit them to the central server. Ongoing support for the community health workers was offered in the form of regular follow-up visits by the research team and troubleshooting support. In-service trainings and workshops were also provided to the local primary health care providers about the study.

### Technology

The DREAM-GLOBAL system in Canada comprised a Bluetooth-enabled blood pressure monitor (A&D UA-767PBT) and a BlackBerry Bold (BlackBerry Inc) mobile phone, with the DREAM-GLOBAL mobile app installed. In Tanzania, an Android mobile phone was used. Blood pressure readings from the UA-767PBT blood pressure monitor were transmitted via Bluetooth to the community health worker’s mobile phone and from the mobile phone to a secure central server in Canada. The central server was programmed to assess the blood pressure readings as normal or high. The server was programmed to wait for up to 3 blood pressure readings, for an individual patient, over 5 min. The average of the 3 readings was then calculated by the central server and then transmitted to the patient’s primary health care provider by fax and to the patient’s own phone by SMS. If the blood pressure was high, an SMS text message was sent to the participant, with advice to contact their health care provider over the coming week (see Multimedia Appendix 3 for the list of SMS text messages). The messages were based on the Hypertension Canadian Clinical Practice Guidelines, and they were developed by clinicians with extensive knowledge of hypertension management [10].

### Evaluation

To evaluate components of the technology and processes of this study, both qualitative and quantitative data were collected. Qualitative data were collected from research notes and from key informant interviews and focus groups, including reflective discussion sessions with research and community teams. This focused on the design, the mechanisms, and context of the intervention, attempting to document local knowledge and expertise within the community stakeholders. Information-gathering tools included developing a community profile, an interview guide to facilitate discussion of topics, and a focus group guide to lead the dialogue on community-specific issues related to the intervention [10]. Quantitative evaluation included what information was delivered, as well as the quality and quantity. During the study, there was an evaluation of the acceptability and accessibility to the SMS text messages, the blood pressure measurement process, including data transmission, the impact of the messages on participants, and the impact of the study process on health care providers. After completion of the study, the main outcome was an assessment of impact on blood pressure control and change of knowledge from the SMS text messages [16]. We have published on the formative data, with early community engagement and readiness [9], and the SMS development process [17]. The change of knowledge is being assessed, and it will be in a separate publication. This paper focuses on the participant, community, and health care provider response to the study processes and technology and the fidelity and quantity of the transmission of study data.

To evaluate the effectiveness of the system and solicit feedback on the technology and study implementation, survey/questionnaires, including simple short answer questions regarding their experiences with the technology, were provided to participants, including community health workers. The questionnaires were designed separately for participants, health care providers, and community leadership. The Participant Evaluation Form for SMS text messages and consent for this evaluation were administered by either the community health worker or the home and community care nurse for that community or the DREAM-GLOBAL clinical coordinator on visits to the communities. Participants were invited to do this feedback, and they provided additional consent to share their responses. Participants were contacted by SMS text messages, by direct phone, or contact through family members to come to the health center for this poststudy evaluation. In total, 184 of 243 participants returned for this final close-out visit. To reinforce the importance of maintaining health information privacy, information was provided at the start of the study, when the general confidentiality form was signed, reviewing that none of their personal information would be disclosed without written consent to anybody other than an investigator, a potential investigator, the client, and an applicable independent ethics committee review board.

## Results

### Participant Feedback

In Canada, the blood pressure lowering study was carried out with 6 First Nations communities in 3 geographic regions, including Manitoulin Island (Ontario), James Bay coast (Quebec), and the north shore of New Brunswick. In Tanzania, a preliminary portion of the study was carried out in 2 rural villages in Hai District, Kilimanjaro Region, to demonstrate that blood pressure can be measured and transmitted through the system. In Canada, a total of 2818 patient blood pressure readings from 243 patients were transmitted to the central server between February 2014 and February 2017. In Tanzania, with the pilot blood pressure measurement study, 1165 readings from 130 patients were transmitted to the central server between October 2014 and August 2015. The study server was programmed to wait for up to 3 readings and then send the mean to the participant’s own phone. This also provided a record about the participant’s blood pressure and allowed the participant to confirm that it was the measurement just taken. There were no incidents of participants finding incorrect readings transmitted from, for example, the participants who had their blood pressure measured immediately before the study. Participants overwhelmingly agreed that the program should be recommended to a friend or relative. Following the study, after presentation of the study progress, all 6 communities supported continuing the program. Only 20 of these participants did not have any blood pressure readings beyond 2 months of enrolling into the study.

An evaluation form was provided with open-ended questions. The results of the Participant Evaluation Form for SMS text messages are found in Table 1.

On the questionnaire, 168 of 187 (89.8%) participants responded that the messages were clear and felt that they made sense. For 137 of 187 (73.2%) participants, the timing and frequency were satisfactory, supporting the process of sending messages on Mondays and Thursdays at 11 am. In response to a probe about privacy concerns about having their blood pressure readings and other health messages sent to their phones, 92 of 121 respondents (76.0%) felt that this was not a problem. Behavior changes noted by participants attributed to the SMS text messages included increased exercise, diet changes to give up sweetened beverages, more awareness of sodium and reading product labels, smoking cessation, and appreciation of the stress reduction messages. In 1 community, the mental health worker shared that their clients were more accessible and receptive if they were participating in the study. Participants also expressed that they felt supported, particularly if they were taking medications and had a greater understanding. Those not on medication noted that they would be more comfortable starting therapy if necessary. Almost one-third of participants shared the messages with family and friends and looked at the texts at their convenience.

An early complaint from the mobile phone app users was the potential to keep the last participant’s name open on the app, when the next participant was ready to have their blood pressure assessed. This would lead to the next participant’s blood pressure being assigned to the previous participant, and it became apparent when the next participant did not receive a confirmatory SMS text message with the blood pressure reading. To address this, the instructions on how to use the app when measuring patients one after another were revised, and the users were updated on the new process. The most frequent complication was a failure to transmit readings by Bluetooth transmission from the monitors to the BlackBerry phones. This was found to be because of the BlackBerry operating system software in older devices, which did not update automatically. This required resetting the device. Some noted that the BlackBerry mobile phone’s version of the DREAM-GLOBAL app was not user friendly—the BlackBerry screens were small, and some parts of the app were difficult to access. In addition, occasional failure of BlackBerry function because of extreme cold or trouble with battery charge was observed. In the early months of study implementation, it was discovered that some BlackBerry devices had not been configured with the correct access point name (APN) setting for the service provider; APN settings were then reconfigured correctly through a troubleshooting process. For Tanzania, none of these problems occurred with the Android software and devices. Updates to the Android software and larger mobile phone screens made it easier to see the roster of participants. More space was allocated for participant’s names to make it easier to distinguish among participants with similar names. When a participant’s phone was replaced, the new number had to be input into the registration software on the Web to ensure the participant continued to receive SMS text messages.

**Table 1:** Evaluation questions: technology and processes.

Target	Intervention	Technology
Patients	Can the patient use the technology, affordability, accessibility?; Is the server/software successfully sending the correct messages?; Training and support required	Fidelity, dose, reach; Recruitment and retention of patients
Providers: community health workers, doctors	Communication and collaboration between health staff and research team; Patient enrollment; Management of hypertension according to clinical practice guidelines?	What training and support were required?; Was study information making it to the patient chart?
Community	Are the communities engaged in the research?; Awareness of stakeholders with the study and willingness to promote	Do the communities have sufficient technology and bandwidth?

### Community Health Workers

The community health workers were asked to provide feedback on their overall experience with the program, from site training to the use of technology. Data are summarized in Table 2.

The community health workers and nurses noted that they were seeing their clients participating in the study more frequently and that they became more comfortable with blood pressure measurement. In addition, participants knew their blood pressure numbers and were better able to address other health care issues, particularly related to mental health issues. They felt that the program led them to have better communication with their physicians and that they had greater confidence about the appropriate therapy for hypertension and knowing when action was needed. They also expressed appreciation for the site visits and ongoing telephone conference communication follow-up. The technological training was well received by the community health workers; in follow-up interviews after the training, almost all either agreed or strongly agreed that the training was helpful and gave them confidence in their ability to perform their role. The community health worker feedback from the site initiation and training is summarized in Table 3 (from 11 community health workers trained across the 6 communities).

After completion of the study, site visits with band leadership and community health leadership and elders were conducted to assess interest and for approvals to continue the program. A total of 5 of the communities remained engaged in the research. A total of 1 community did not allow follow-up with the community leadership during the study, but it did welcome a poststudy review and supported continuing the program after the study completion.

Health care providers shared that they noted that their patients participating in the study appreciated being contacted on their cell phones by the SMS text messages. They felt that their patients were more informed and engaged in their disease management and more likely to follow-up with their physicians. Many were surprised to see that their patients were calling them and making appointments, as per the instructions from their cell phone. It was noted that the white coat effect for blood pressure was identified through the community measurement of blood pressure, and more people, who would otherwise not visit, visited the health center.

**Table 2:** Results of participant evaluation for SMS text messages.

Questions and answers		Values, n (%)
**Were the messages you received clear enough? Did they make sense to you? (N=187)**		
	Yes	168 (89.8)
	No	19 (10.1)
**Were the timing and frequency of messages okay for you? (N=187)**		
	Yes	137 (73.2)
	No	42 (22.4)
	—^a^	8 (4.2)
**Was your experience with the phone positive? (N=179)**		
	Yes	102 (56.9)
	No	36 (20.1)
	—	41 (22.9)
**Did you have any concerns about receiving health messages on your phone (ie, privacy/confidentiality)? (N=121)**		
	Yes	7 (5.7)
	No	92 (76.0)
	—	22 (18.1)
**What did you like best about receiving SMS text messages? (N=144)**		
	Information/content	68 (47.2)
	Motivation/reminders	43 (29.8)
	Easy to read and understand	19 (13.1)
	Useful/came in handy	10 (6.9)
	Felt cared for	4 (2.7)
**What did you like least about receiving text messages? (N=52)**		
	Messages too repetitive	20 (38.4)
	Disliked content	11 (21.1)
	Could not use phone	8 (15.4)
	Messages too long	6 (11.5)
	Messages too frequent	3 (5.7)
	Generic messages	2 (3.8)
	Text was too small	2 (3.8)
**Would you recommend participating in this program to a friend or relative with hypertension? (N=169)**		
	Yes	165 (97.6)
	No	3 (1.7)

^a^Not applicable or participants did not receive messages.

**Table 3:** Community health workers’ feedback from site training and initiation of study (N=11).

Community health workers’ excerpts		Strongly disagree, n (%)	Disagree, n (%)	Neutral, n (%)	Agree, n (%)	Strongly agree, n (%)
**Uptake of knowledge**						
	I now have a good understanding of my role in the DREAM-GLOBAL^a^ study	0 (0)	0 (0)	0 (0)	4 (36)	7 (64)
**Uptake of skills**						
	I feel confident that I will be able to explain DREAM-GLOBAL to community members	1 (9)	0 (0)	1 (9)	4 (36)	5 (46)
	I feel confident in how to use the Manual of Operations for screening and enrolling patients	0 (0)	0 (0)	0 (0)	7 (64)	4 (36)
	I feel confident in how to identify, enroll, and register participants into the study	1 (9)	0 (0)	1 (9)	5 (46)	4 (36)
	I feel confident in using Canadian Hypertension Education Program (Hypertension Canada) guidelines to take and assess blood pressure readings	0 (0)	0 (0)	0 (0)	6 (54)	5 (46)
	I feel confident that I will be able to take and submit blood pressure readings using the BlackBerry device	0 (0)	0 (0)	1 (9)	6 (55)	4 (36)
	I feel that I know how to use the safety protocols and process for submitting case report forms	0 (0)	0 (0)	0 (0)	8 (73)	3 (27)
	This training was a good way to exchange information and learn about the DREAM-GLOBAL study	1 (9)	0 (0)	0 (0)	2 (18)	8 (73)

^a^DREAM-GLOBAL: Diagnosing hypertension—Engaging Action and Management in Getting Lower Blood Pressure in Indigenous and low- and middle-income countries.

### Community

Overall, despite some initial issues, the technology performed as hoped in delivering the SMS text messages to patients, which garnered a positive reception from the communities involved. It also demonstrated that communities had enough technological resources to manage the study. The fact that the community health worker time to manage the study in each community was granted by the health leadership indicates the commitment and engagement of each community. All communities involved requested to have the program continue after the study’s completion.

## Discussion

### Principal Findings

DREAM-GLOBAL was designed to increase the capacity for affordable, evidence-based, guidelines-driven hypertension management interventions at the patient, provider, and community level. This study demonstrated that information could be obtained and transmitted with fidelity from participants in remote settings. It also confirmed that patients were willing and able to participate in the program [18]. It was also possible for community health care providers, including nonmedical health workers, to train study participants to successfully use the technology. The pairing of Bluetooth and wireless technology enabled the secure, reliable, and widespread transmission of information from participants to their health care provider, including the transmission of guideline-based SMS text messages to study participants.

### Insights

Community health workers and professional health care providers found that the technology was able to enhance their communication with participants and between each other. The process assisted with participant engagement in their own health care, which has also been demonstrated [19]. Training and support requirements were pragmatic. With achievement of improved blood pressure control, there is at least indirect evidence that study information was positively impacting on participant health care [16]. A theme emerging from community health workers was that the DREAM-GLOBAL technology could be more user friendly. For example, the Blackberry operating system was outdated and often had to be reset. The Android mobile phones and Android app used in Tanzania had much fewer problems. The BlackBerry mobile phone even failed to work in extreme cold weather.

The DREAM-GLOBAL system used minimal resources, requiring only the server, devices, and bulk SMS transmission. Transmission of blood pressure data has also recently been validated [20]. This system can easily be scaled to additional participants, with minimal additional costs. Assignment of blood pressure measurement to the community health worker addresses the realities of health care worker shortages in rural and remote communities. Shifting measurement from a health care provider to community health workers is a way to strengthen and expand community resources. The technology therefore linked the community health center, community health worker, home and community care nurses, and local physicians, thus supporting members within the circle of care.

### Conclusions

The DREAM-GLOBAL study has resulted in an innovative system that has the ability to provide blood pressure screening and connect patients to health care providers in the community who can diagnose and treat hypertension. The technical platform provided a solution for hypertension management in low-resource settings, with the use of task shifting in blood pressure measurement and meaningful SMS text messages that promote health behavior change. The system has the potential to further improve patient care, as it can be easily adapted to regional needs and scaled up to include other aspects of chronic disease management.

## Appendix

Multimedia Appendix 1 Circle of care diagram for Diagnosing hypertension—Engaging Action and Management in Getting Lower Blood Pressure in Indigenous and low- and middle-income countries.

Multimedia Appendix 2 Combined training documents for Diagnosing hypertension—Engaging Action and Management in Getting Lower Blood Pressure in Indigenous and low- and middle-income countries.

Multimedia Appendix 3 Diagnosing hypertension—Engaging Action and Management in Getting Lower Blood Pressure in Indigenous and low- and middle-income countries short message service text messages.

Multimedia Appendix 4 CONSORT-EHEALTH checklist (V 1.6.1).

## Abbreviations

APN: access point name
DREAM-GLOBAL: Diagnosing hypertension—Engaging Action and Management in Getting Lower Blood Pressure in Indigenous and low- and middle-income countries
LMICs: low- and middle-income countries
