# Supplementary figures and images for: Analysis of the Implementation, User Perspectives, and Feedback From a Mobile Health Intervention for Individuals Living With Hypertension (DREAM-GLOBAL): Mixed Methods Study
Source: JMIR Mhealth Uhealth. 2019 Dec 9;7(12):e12639. doi: 10.2196/12639 (PMC6928701; doi:10.2196/12639)

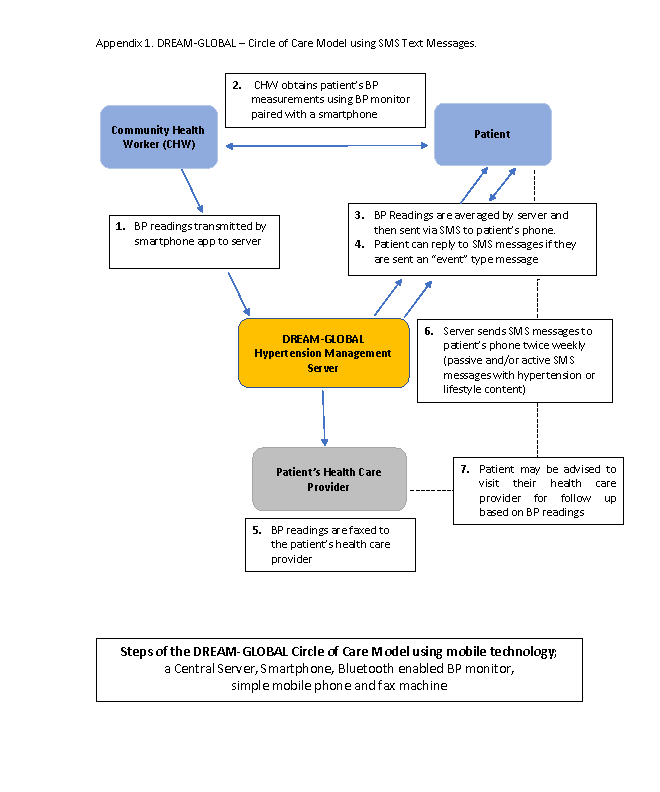

Supplement: Multimedia Appendix 1 [file mhealth_v7i12e12639_app1.png]
