# Supplementary material for: Analysis of the Implementation, User Perspectives, and Feedback From a Mobile Health Intervention for Individuals Living With Hypertension (DREAM-GLOBAL): Mixed Methods Study
Source: JMIR Mhealth Uhealth. 2019 Dec 9;7(12):e12639. doi: 10.2196/12639 (PMC6928701; doi:10.2196/12639)
Supplement: Multimedia Appendix 2 [file mhealth_v7i12e12639_app2.pdf]

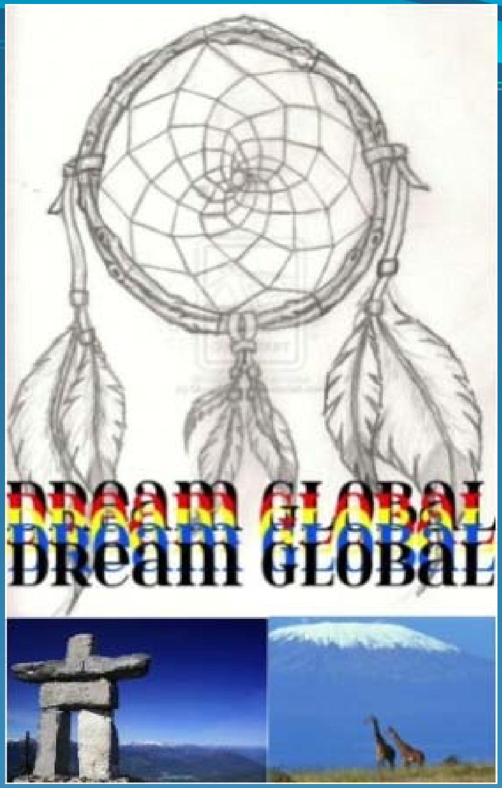

DREAM-GLOBAL
Short Message Service
SMS Text Messages

1. Background

- DASH diet recommendations for heart health were also consulted
- Original messages developed based on clinical practice guideline by physicians
- Decision was made to pilot test the messages for the best "fit" with community members and to make them culturally appropriate and culturally safe.

2. Development of SMS messages

- DG team members conducted Focus Groups in three First Nation communities
- Focus Group members gave their comments on the messages: the content, the "tone" and the language
- All comments were compared, and messages were changed to respond to comments.
- Result: some messages cut, some language changed because it was too scary, some messages made more positive in tone; more concrete examples, and some traditional foods included.

3. Cell Phone SMS Text Description of Active Messages

ACTIVE Type	Canada/English
Measurement normal	You are doing a good job of managing your blood pressure! Your blood pressure is **s/d** today.
Measurement high	Your blood pressure is **s/d** today, not quite at target. Please talk to your health care provider about what to do.
Measurement very high	Your blood pressure is **s/d** today – this is very high. Please see your doctor or health provider as soon as possible
Physiology/Importance of HTN Therapy	Taking your medication eases the stress on your heart and lowers your blood pressure, reducing the chance of strokes.
Risk/Benefit Side Effects	The medications provide many benefits but might have side effects. Talk to your health care provider if you have any side effects.
Drug Treatment	If you are taking your blood pressure pills according to instructions, you will get the best results.
Drug Classes for Multiple Drug Therapy	Your doctor might prescribe more than one kind of blood pressure pill for you.
Drug Classes for Multiple Drug Therapy	Most people need more than one kind of pill to control their blood pressure
Adherence	Have you taken your medication today? Your medications work much better if you take them every day at the same time
Adherence	Your blood pressure pills can be taken together.

4. Event Messages

Health Care Provider - Event	This is a reminder to see your health care provider within 14 days.
EVENTS	A friendly reminder: Have you seen your health care provider in the past 7 days? 1 = yes 0 = no

5. Passive Messages

PASSIVE Type	Canada/English
Stress	Relaxation techniques like deep breathing help you control your blood pressure.
Sodium	Cooking with less salt or no salt will help to lower your blood pressure.
Sodium	For a healthier blood pressure, do your best to reduce or avoid processed meats like bacon, bologna, salami and sausage.
Sodium	You can reduce the salt in canned foods like beans, peas or tuna with water, by rinsing them before using.
Sodium	Choosing foods where the label shows less than 15% of the Daily Value for sodium is one way to help lower your blood pressure.
Sodium	Choosing foods that say “sodium-free”, “low sodium” or “no added sodium” will help you have a healthier blood pressure.
DASH - Fruit	Try to eat fruit more often as a snack or with meals.
DASH - Vegetables	Try to increase the amount of vegetables you eat every day by having a serving at lunch and another at dinner.

Passive Messages – Dietary Choices

DASH - Lean Meats & Poultry	When eating meat, a healthy portion is about the size of the palm of your hand.
DASH - Lean Meats & Poultry	Try meatless meals a couple of times a week, using beans or lentils, because they have less unhealthy fat.
DASH - Lean Meats & Poultry	Did you know that you can trim away the skin and fat from poultry and meat to make it healthier for your heart?
DASH - Lean Meats & Poultry	A heart healthy diet includes at least a couple of servings of fish every week, especially local fresh fish.
DASH - Lean Meats & Poultry	A heart healthy diet uses lean meats like moose, deer, caribou, lean beef and pork, and poultry with the skin removed.
DASH - Lean Meats & Poultry	Healthy cooking tip: there are lots of ways to flavour food without using salt - like onions, herbs, spices, vinegar, garlic, ginger, lemons, sodium-free broth, or a bit of reduced-sodium soy sauce.

Passive Messages-
Dietary choices and Dining out

DASH - Nuts, seeds	Nuts provide fiber and minerals that are heart healthy. A healthy serving is ¼ to 1/3 cup, without added salt.
DASH - Sweets & Alcohol	It is good for your heart if you cut back on foods with lots of sugar that give you too many calories, such as pop, candy, cookies and sweets.
DASH - Sweets & Alcohol	A heart healthy diet limits alcohol to two drinks or less each day for men and one drink or less each day for women.
DASH - Dining out	Tip: For a healthier heart, don't use the salt shaker.
DASH	When shopping, take a minute to read nutrition facts on the label, so you can choose foods lower in sodium, sugar and fat.

Passive messages – Activities of Daily Life

Physical Activity	Regular physical activity can lower your blood pressure. Aim for at least 30 minutes most days of the week of moderate physical activity such as walking.
Physical Activity	Walking, snowshoeing, canoeing, swimming or fitness classes all help lower blood pressure when done regularly.
Physical Activity	Don't be afraid to be active. If you have not been active for quite some time or if you are beginning a new activity or exercise program, take it gradually
Physical Activity	Regular physical activity makes your heart stronger, so it can pump more blood with less effort, lowering your blood pressure.
Physical Activity	For some people getting regular exercise is enough to reduce blood pressure.
Physical Activity	Regular exercise helps you maintain a healthy weight and also helps to control blood pressure.
Smoking	Reducing tobacco use is good for your heart and blood pressure, because the heart rate will be healthier and the heart will get more oxygen.
Smoking	Quitting smoking is the most powerful thing you can do to improve your health.

DREAM – GLOBAL STUDY

BP Technology Training Session

February 2014

OUTLINE

1. DREAM-GLOBAL: Enhancing the ‘Circle of Care’
2. Preparing to See Your Patients
3. About your BlackBerry Device
4. About your Blood Pressure Monitor
5. What to do about problems: Troubleshooting Tips
6. Contact Information
7. Demo – Return Demo
8. Meegwetch!

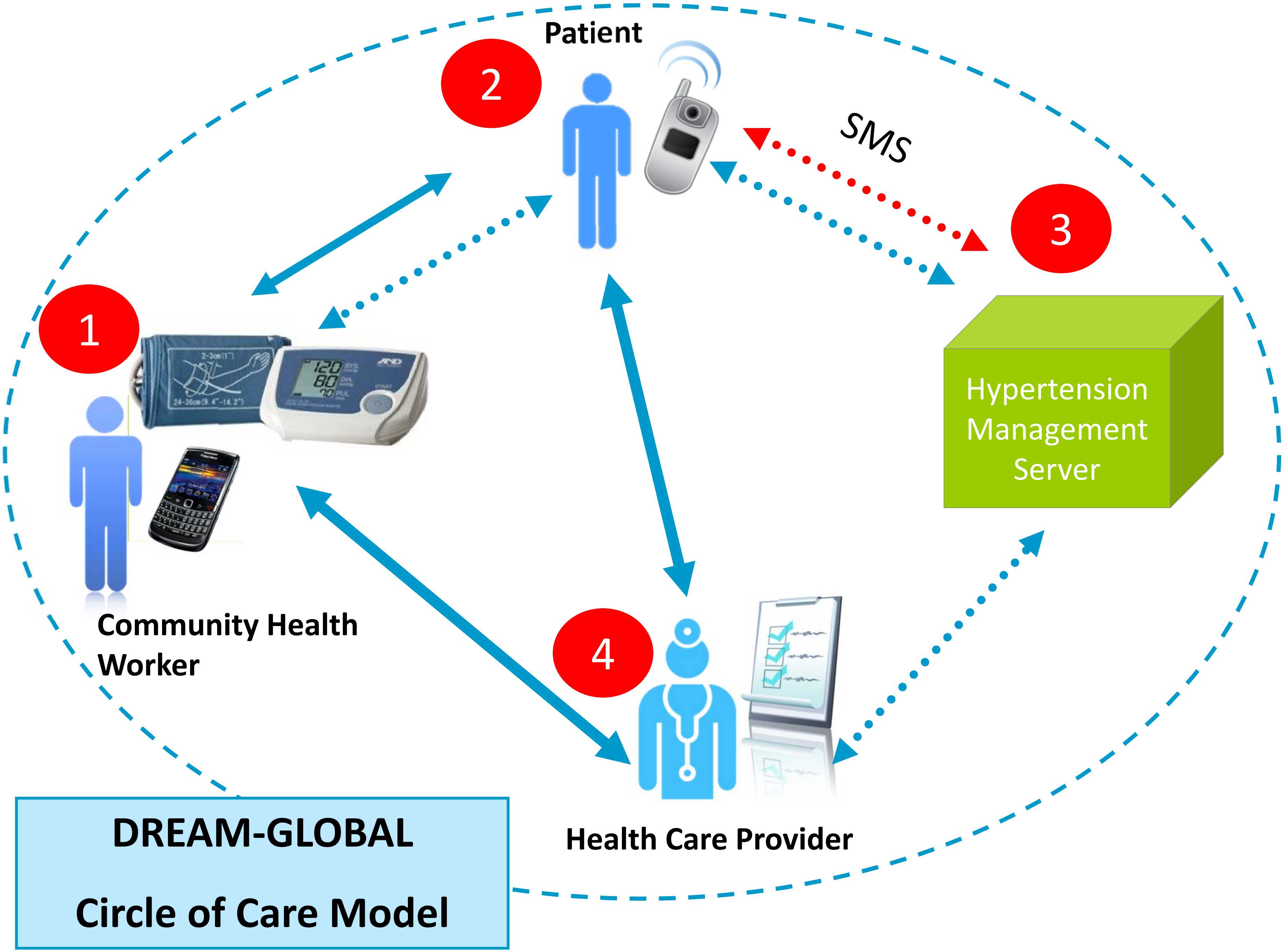
Patient
SMS
1
2
3
4
Hypertension
Management
Server
Community Health
Worker
Health Care Provider
DREAM-GLOBAL
Circle of Care Model

Preparing to see Patients

Community Health Worker

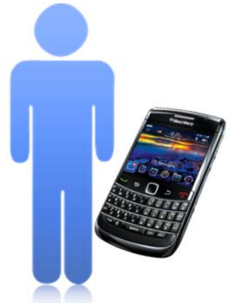

1 Recharge your phone before seeing patients

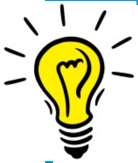

RESET: If your phone is **not** working, remove the battery and put it in again

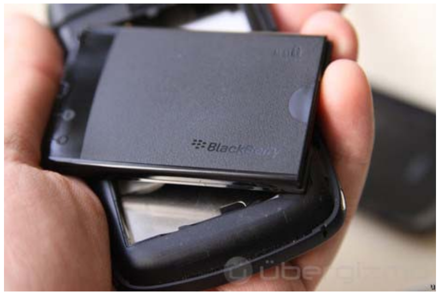

2 Lock your phone when you are not using it (Press LOCK button)

3 Is your phone fully charged?

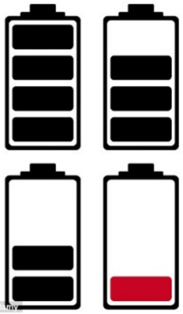

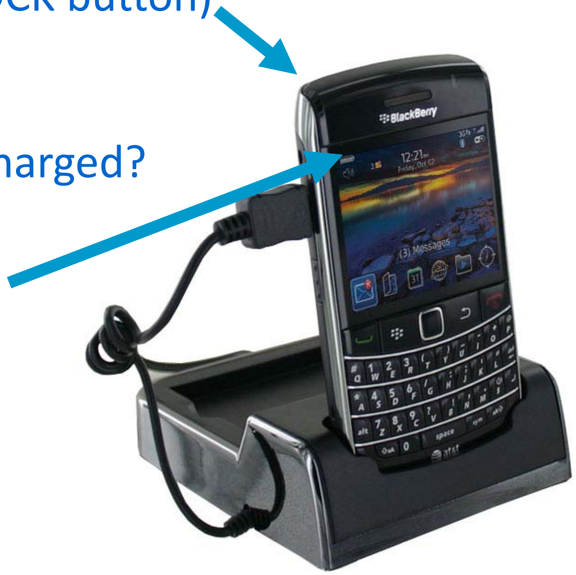

4 Use the BlackBerry and correct blood pressure monitor together

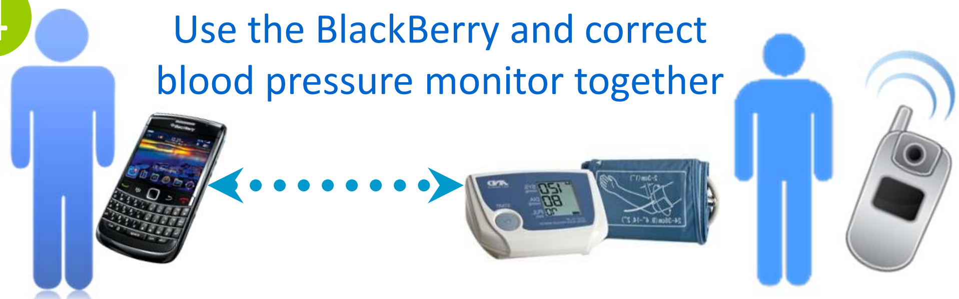

Community Health Worker **Patient**

A Closer Look at Your **BlackBerry Device…**

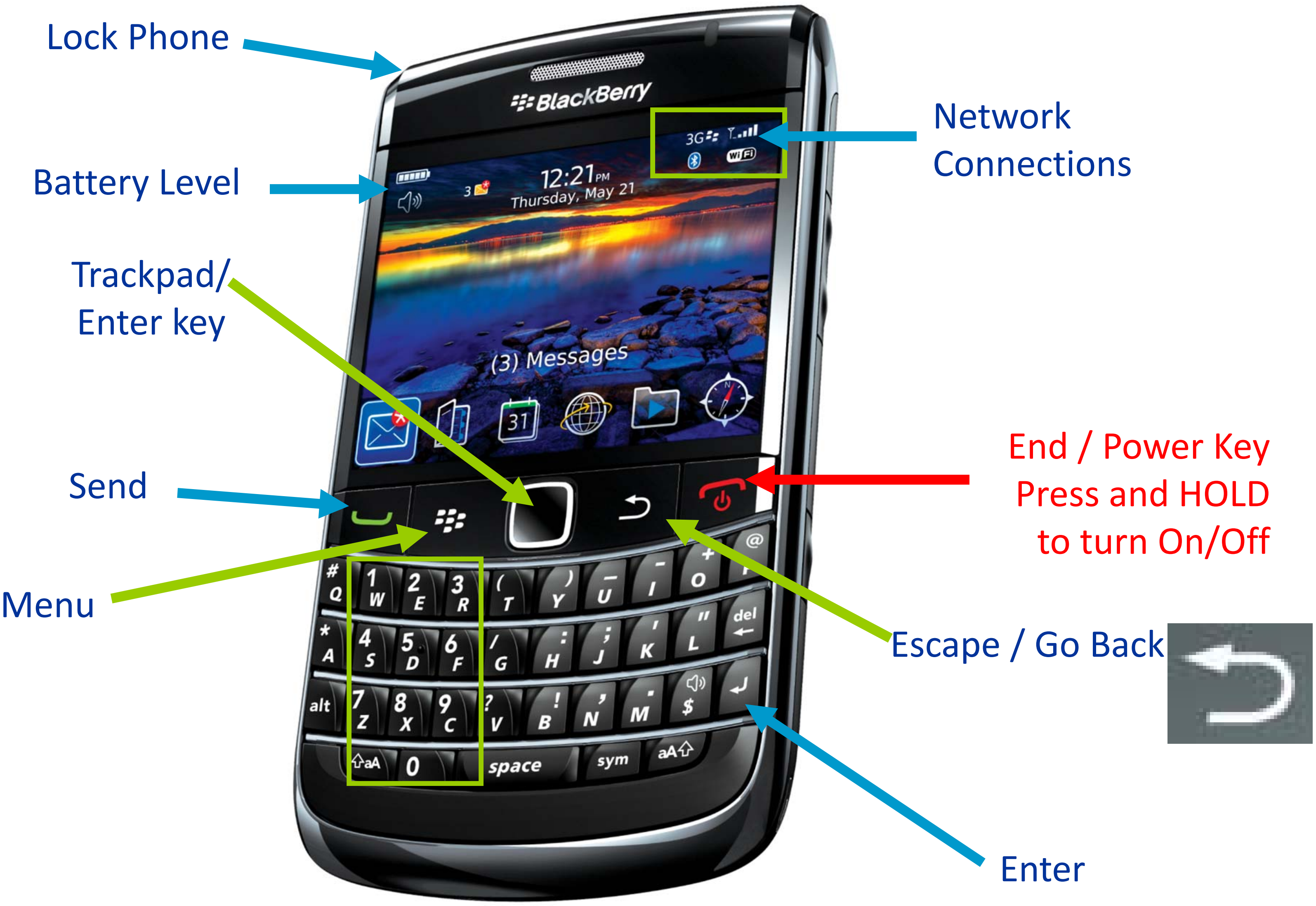

Device: Blackberry 9700

BlackBerry Bold 9700 Smartphone

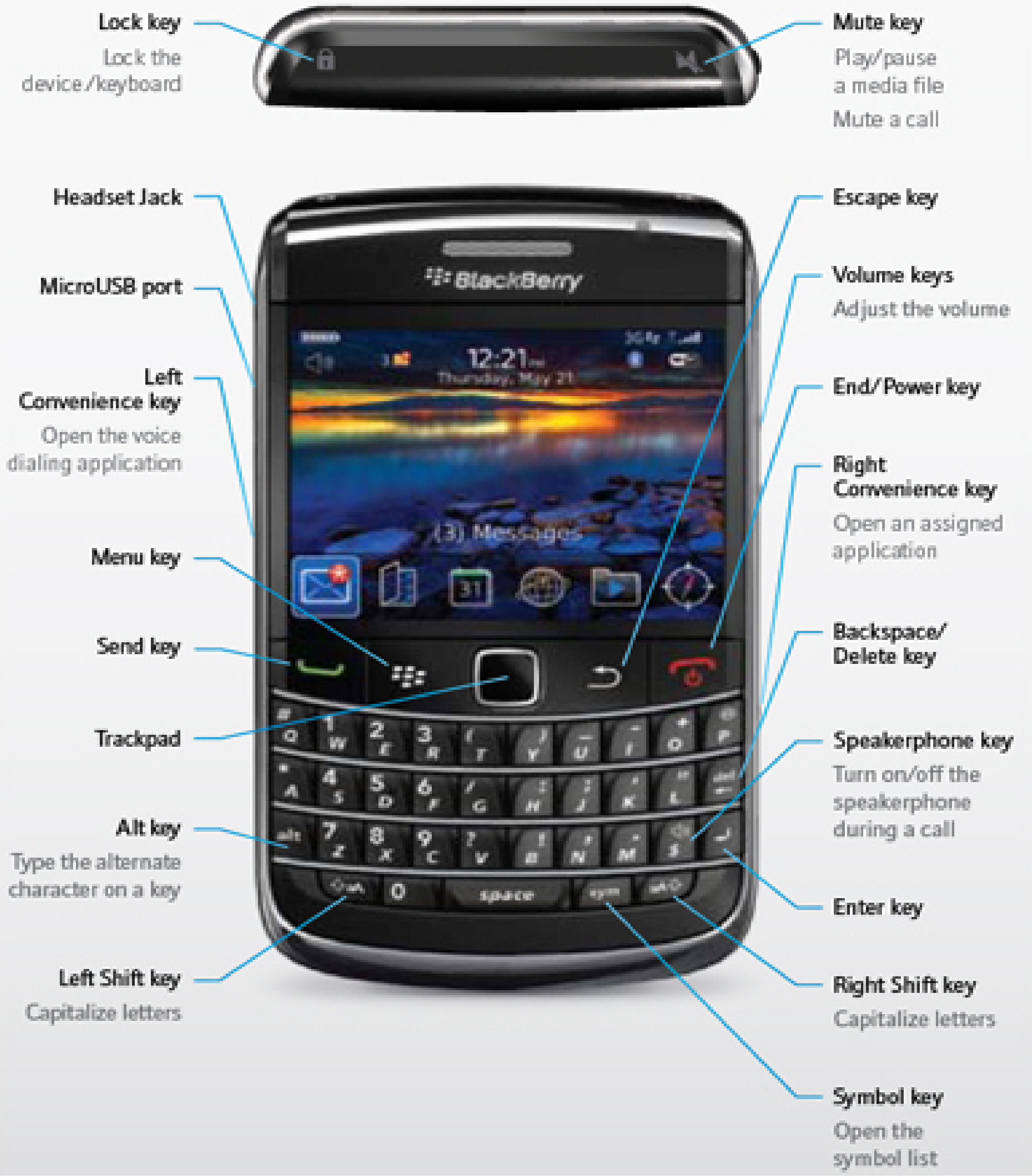

A Closer Look at Your **Blood Pressure Monitor**…

Set Up: Taking a Blood Pressure Reading

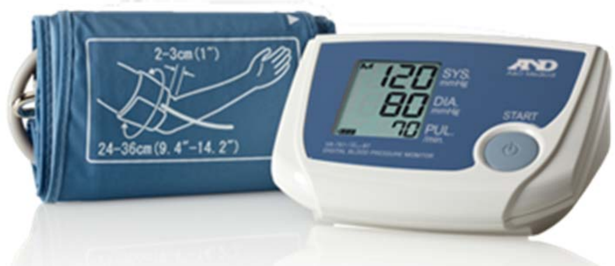

Model UA-767 *Plus*BT

Connecting The Air Hose

Insert the air connector plug into the air socket firmly.

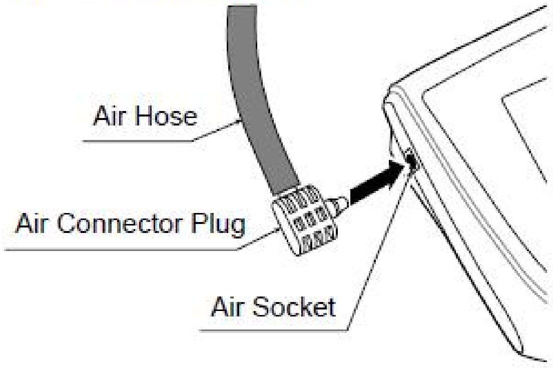

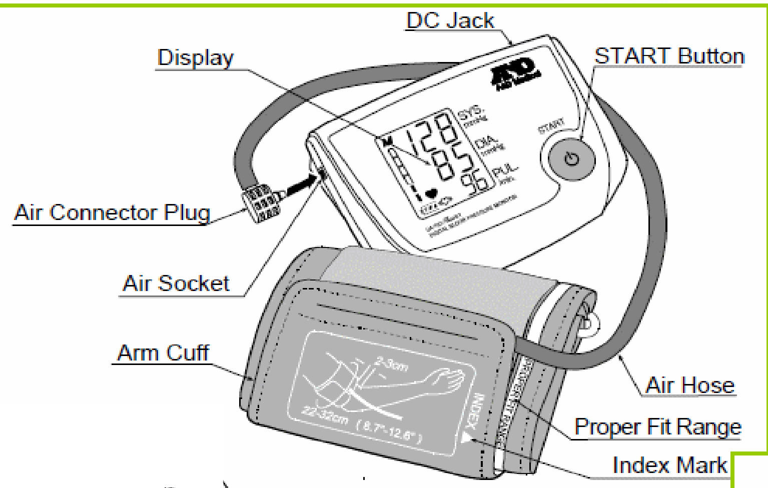

Part of Display

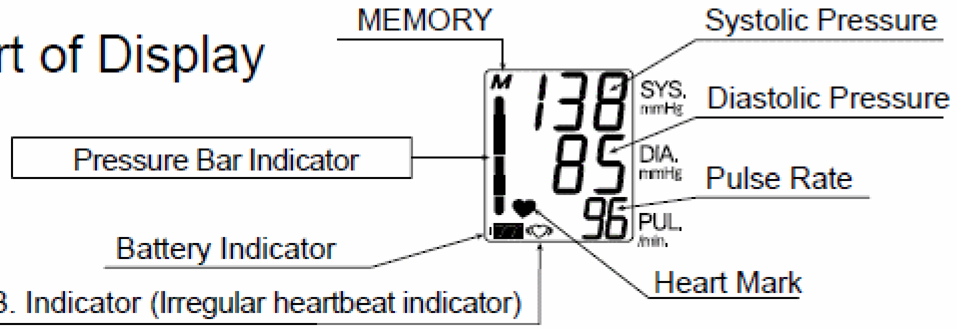

Measurement With The Desired Systolic Pressure

If your systolic pressure is expected to exceed 230 mmHg or you use the optional small cuff, use this procedure.

1. Place the cuff on the arm (preferably the left arm).
2. Press and hold the START button until a number about 30 to 40 mmHg higher than your expected systolic pressure appears.
3. Release the START button to start measurement, when the desired number is reached. Then continue to measure your blood pressure as described on the previous page.

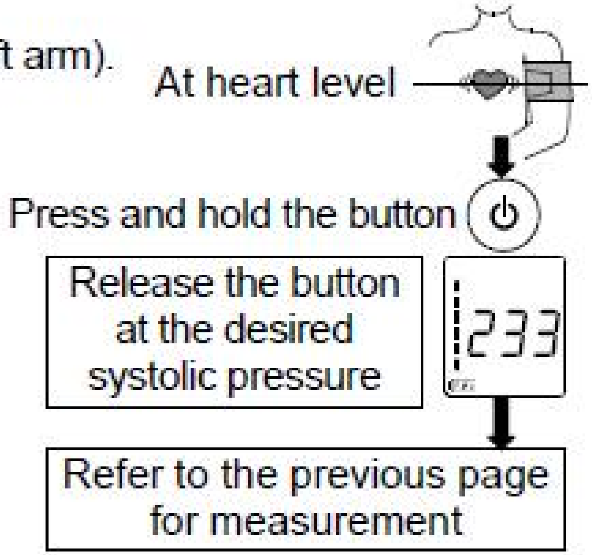

Replacing Batteries

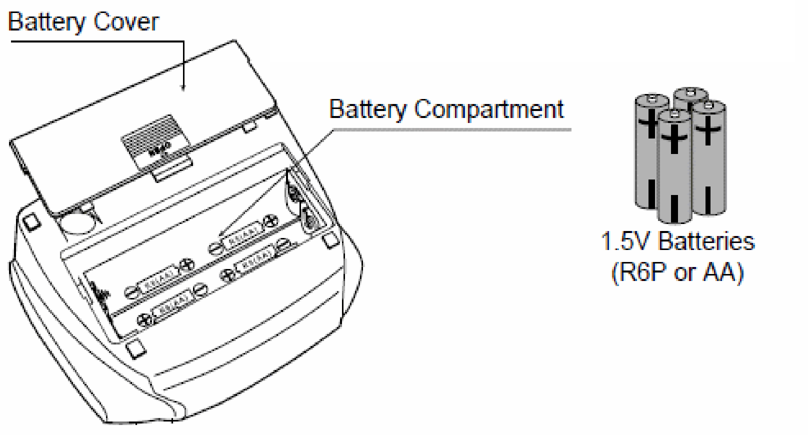

Installing / Changing The Batteries

1. Slide the battery cover up to open it.
2. Remove the used batteries and insert new batteries into the battery compartment as shown, taking care that the polarities (+) and (-) are correct.
3. Slide the battery cover down to close.

Use only R6P, AA batteries.

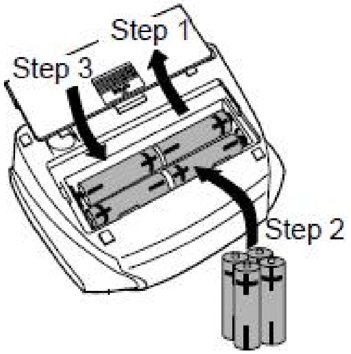

CAUTION

- ❑ Insert the batteries as shown in the battery compartment. If not, the device will not work.
- ❑ When (LOW BATTERY mark) blinks in the display, replace all batteries with new ones. Do not mix old and new batteries. It may shorten the battery life, or cause the device to malfunction.
- ❑ (LOW BATTERY mark) does not appear when the batteries are drained.
- ❑ Battery life varies with the ambient temperature and may be shorter at low temperatures.
- ❑ Remove the batteries if the device is not to be used for a long time. The batteries may leak and cause a malfunction.
- ❑ Use the specified batteries only. The batteries provided with the device are for testing monitor performance and may have a limited life.

Set Up: Taking a Blood Pressure Reading

1

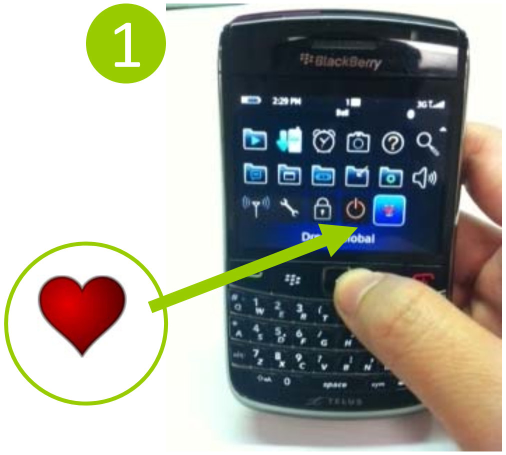

Click on the DREAM-GLOBAL application

Bluetooth

Q: How are readings transmitted from the BP monitor to the phone?

A: The BP readings will be sent automatically to the phone if they are close to each other through Bluetooth transmission

2 Set up the BP cuff and monitor, fit the cuff on your patient.

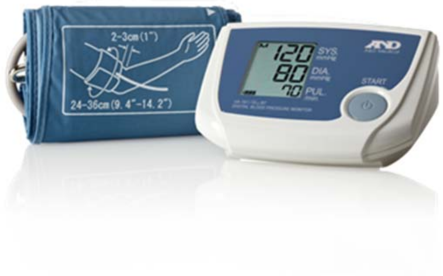

3 Choose your patient from the list

4 The app will say: ***Please take a reading.*** *(Remember to follow CHEP Guidelines)*

5 Take 3 readings. Write down each reading before starting a new one.

6 ***Accept and send*** BP readings that match those that you wrote down. The readings will be sent to the server.

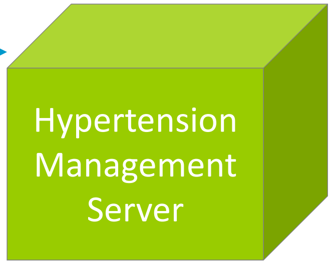

Review of STEPS

1. Click on the Dream Global App on the BlackBerry
2. The App will list patient names. Click on the patient you want.
3. When instructed, take a BP reading.
4. Repeat for 3 readings total.
5. When asked, click to accept BP readings.
6. Follow instructions to accept BP readings and send to the server.
7. To take a new BP reading for another patient, restart the app or go back to the list of patients

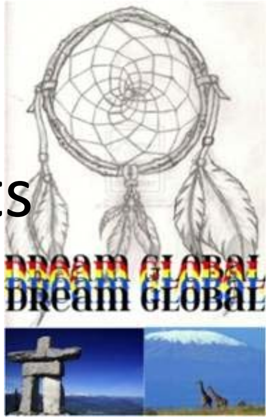

Symbols	Function / Meaning	Recommended Action
⏻	Standby and power on.	————
⊕ R6(AA) ⊖	Battery installation guide	————
⎓	Direct current	————
SN	Serial number	————
2006	Date of manufacture	————
	Type BF: Device, cuff and tubing are designed to provide special protection against electrical shocks.	————
♥	The indicator that appears while measurement is in progress. It blinks when the pulse is detected.	Measurement is in progress. Remain as still as possible.
((♡))	**I**rregular **H**eart**b**eat indicator. (I.H.B.) The indicator that appears when an irregular heartbeat or any excessive body movement is detected during the measurement.	————
M	Previous measurements stored in MEMORY.	————
Full Battery	The battery power indicator during the measurement.	————
Low Battery	The indicator blinks when battery power is low.	Replace all batteries with new ones, when the indicator blinks.
Err	Unstable blood pressure due to movement during the measurement.	Try the measurement again. Remain very still during the measurement.
	The systolic and diastolic values are within 10 mmHg of each other.	Fasten the cuff correctly, and try the measurement again.
	The pressure value did not increase during inflation.	
Err CUF	The cuff is not fastened correctly.	
Err PUL. DISPLAY ERROR	The pulse is not detected correctly.	
SYS	Systolic blood pressure in mmHg	————
DIA	Diastolic blood pressure in mmHg.	————
PUL./min	Pulse per minute	————

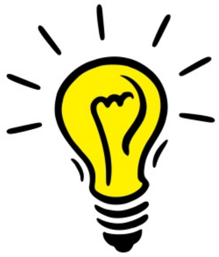

HELP

TROUBLESHOOTING TIPS

GENERAL TROUBLESHOOTING TIPS

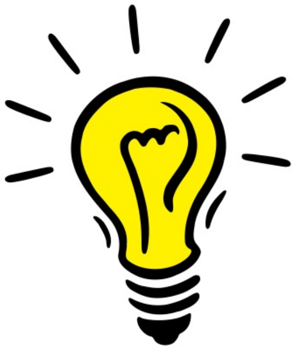

Problem	Possible Reason	Recommended Action
Nothing appears in the display, even when the power is turned on.	Batteries are drained.	Replace all batteries with new ones.
	Battery terminals are not in the correct position.	Reinstall the batteries with negative and positive terminals matching those indicated on the battery compartment.
The cuff does not inflate.	Battery power is low. (LOW BATTERY mark) blinks. [If the batteries are drained completely, the mark does not appear.]	Replace all batteries with new ones.
The unit does not measure. Readings are too high or too low.	The cuff is not fastened properly.	Fasten the cuff correctly.
	You moved your arm or body during the measurement.	Make sure you remain very still and quiet during the measurement.
	The cuff position is not correct.	Sit comfortably and still. Raise your hand so that the cuff is at the same level as your heart.
	———	If you have a very weak or irregular heat beat, the device may have difficulty in determining your blood pressure.
Other	The value is different from that measured at a clinic or doctor's office.	See "Why measure blood pressure at home".
	———	Remove the batteries. Place them back properly and try the measurement again.

GENERAL TROUBLESHOOTING TIPS

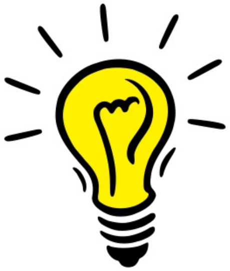

Problem	Recommended Action
BP readings take a long time to show up on the BlackBerry	1. Make sure the BlackBerry screen says, *"Please take a reading…"*<br>2. Wait and be patient. **<u>Or</u>** Take another blood pressure reading.
BP readings take a long time to "send" from the BlackBerry to the server	1. Wait and be patient. **<u>Or</u>** Take out battery and restart device, turn it back on.<br>2. Start the Dream Global App again and see if the readings have been sent.
BlackBerry does not recognize the BP monitor.	If you use a monitor that was not matched to your phone, you will be asked for a code. **Enter Code: 39121440**
The BP monitor is sending too many BP readings.	The BP monitor can store lots of readings. Sometimes there are old readings left on the monitor.<br>Make sure you write down new numbers from your patients after each reading, and '*accept*' and send only the readings that match those of your patients.

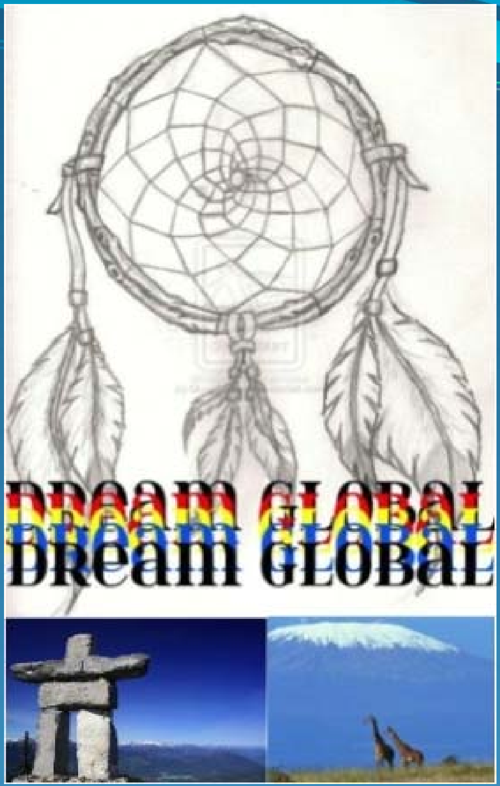

DREAM-GLOBAL
Blood Pressure Assessment

1. What is High Blood Pressure?

- High blood pressure = Hypertension
- Blood pressure changes throughout the day depending on activity.
- When blood pressure is above the healthy range it is referred to as high blood pressure or hypertension.
- As we age, blood vessels become narrowed and this causes more work for the heart to pump the blood. This causes increased pressure in the blood vessels.

1 in 5 First Nations people in Canada has high blood pressure

What is High Blood Pressure

- High blood pressure is also called the “silent killer”; you cannot feel when you have high blood pressure.
- If your BP remains high for a long time, it damages the blood vessels and can cause them to block, decreasing the blood flow to various parts of the body.
- Think of it like water in a garden hose. As water travels through the hose, it creates pressure against the hose.

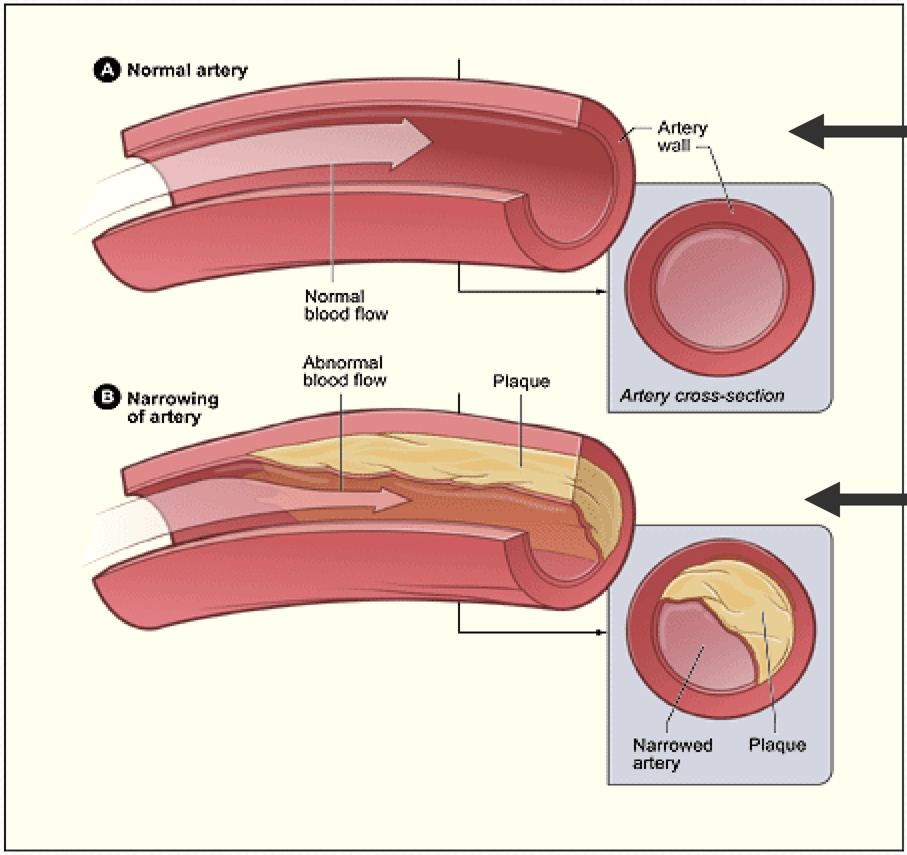

Normal Artery

Plaque Build-Up

Plaque causes narrowing of the artery wall which can result in a rise in blood pressure

2. Target Blood Pressures

- Blood pressure ***greater than or equal to 140/90*** is considered too high when taken in the health care provider's office

- Persons with **diabetes** whose blood pressure is **greater than or equal to 135/85** is considered too high if measured at home

3. High blood pressure can harm the body in the following ways:

- Memory loss or stroke

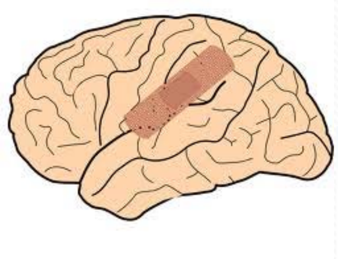

- Angina, heart pain or heart attack

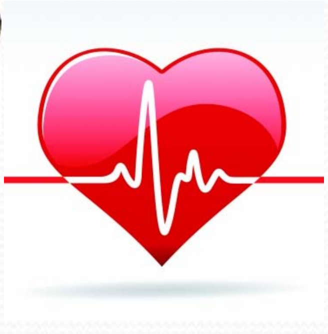

- Kidney damage

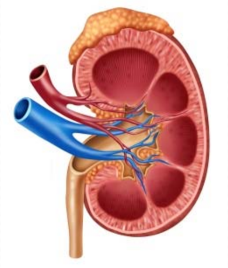

- Impaired vision or blindness

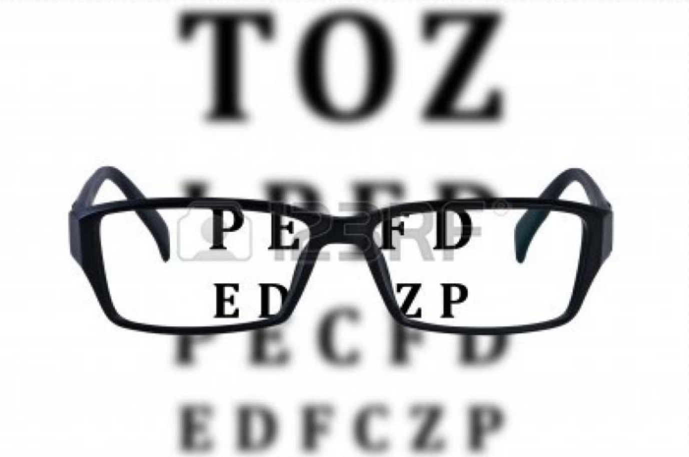

4. Other major risk factors for HTN

**Smoking**	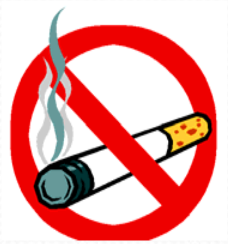
**Uncontrolled diabetes**	HBA1c greater than 0.070
**High cholesterol**	Based on individuals at intermediate risk LDL greater than 3.5 mmol/L

5. How to Monitor Blood Pressure

- Sit client comfortably and quietly, feet flat on the floor and back supported with the arm at heart level.
- Choose the right size of cuff and place on the arm.
- Wait for 5 minutes, then take three readings each 2 minutes apart.
- Copy each reading into the client's log.

Copy each reading into the client's log.

Example:

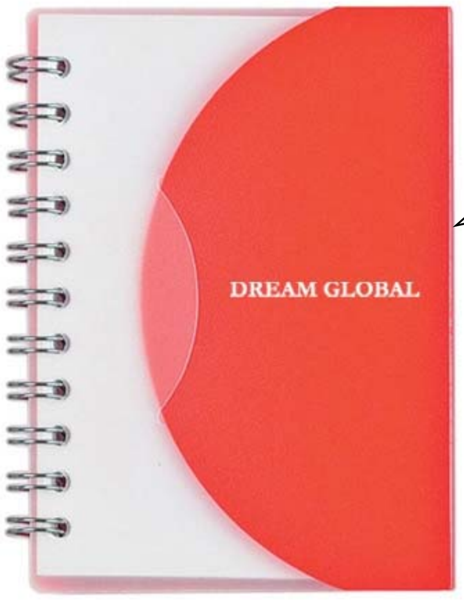

Feb 6, 2014

134 / 85 mm Hg

139 / 88 mm Hg

140 / 89 mm Hg

What do the numbers mean?

140
90

Systolic Blood Pressure (Top Number)

When the heart beats and pumps blood

Diastolic Blood Pressure (Bottom Number)

When the heart relaxes and fills with blood

- When patients have **diabetes**, readings should be **130/80** or less.
- Ideally blood pressure readings should be **120/80** or less.

Blood Pressure Assessment for Dream Global

GUIDE FOR BLOOD PRESSURE ASSESSMENT

Condition	Systolic and Diastolic	Assessment
Uncomplicated < 80 years old	<140 and < 90	**Normal**
Diabetes < 80 years old	< 130 and < 80	**Normal**
Elderly person Age 80 or older	<150 and <90	**Normal**
Uncomplicated < 80 years old	Systolic 140 -179 or Diastolic 90 -109	**High - Instruction:** Please advise patient to talk to their health care provider about what to do.
Diabetes < 80 years old	Systolic 130 -179 or Diastolic 80 -109	**High - Instruction:** Please advise patient to talk to their health care provider about what to do.
Elderly person age 80 or older	Systolic 150 -179 and Diastolic 90 -109	**High - Instruction:** Please advise patient to talk to their health care provider about what to do.
Anyone	Systolic 180 and above or Diastolic 110 and above	**Very High - Instruction:** Please instruct patient to see their doctor or health care provider **as soon as possible.**

For any questions, please contact us at:
Tel: 416-480-6100 x 7064 or Email: dream.global@sunnybrook.ca

Blood pressure workshop

Please choose a partner and let's practice!!

- Practice blood pressure device set up
- Patient positioning and timing or readings
- Recording of blood pressure readings

Who should be trained	Agenda	Method	Tools	Time requirement
CHR	Consent process Good Clinical Practice	interactive workshop	slide set: Informed Consent Workshop/workbooks/worksheet	1.5 hours
CHR	Blood pressure evaluation	hands on/return demo	slide set: Blood Pressure Assessment:CHEP patient slide set, BP levels/ALERTS	1 hour
Nurse Manager	Completion of CRF	direct teaching	Guidelines for completion/Case Report Form worksheet	1 hour
	Technology Cell phone/Blackberry	demo/ return demo	Slide set: BP Technology Training Session	3 hours
	Cell phone safety	hand outs for patients	Cellular Safety / Cell Phone Tips /Study participant ID cards	1/2 hour
Nurse	Study contact hotline	Information sheet	Trial Center File	1/2 hour
	Conference calls format	slide presentation	template for follow up	`1/2 hour
	Troubleshooting technology	demo/ return demo	laminated card (troubleshooting guide Jan 14) / two sided laminate card	1 hour
	Pairing devices	demonstration	return demonstration / interactive	3 hours
	ALERT system for SMS	laminiated card	Guide for Blood Pressure Assessment (figure 2)	1 hour
	Frequency of subject follow up visits	Study Protocol	slide set: Protocol Overview	1 hour
	Practice patient screening/ enrollment process		return demonstration interactive	3 hours
	SMS messages	slide presentation/demo	slide set: SMS Text Messaging /demonstration on BB	1 hour
	Management of documents	screening documents	Trial Center file/ Study Procedures and Administrative Manual	1 hour
Physician	role of physician for study participants			
Physician	respond to ALERTS			
Physician	engagement with CHR/NP			
Physician	Fax notification process			
Office Manager	scheduling study patients on an emergent basis			

STEP BY STEP: HOW TO TAKE AND SEND BP READINGS

1. Set up the BP monitor and cuff to fit your patient's arm.
2. Click on the Dream Global App on your BlackBerry to start.
3. Select your patient from the list. Click "Next" to go to next screen.
4. Take a BP reading by pressing START on the monitor. Write down each reading from the monitor's display into your worksheet.
5. Click "Yes" to Accept readings to send them to the server. **Reminder:* always check that you are sending the correct readings to the server. Click "No" for readings that do not match your patient.
6. Repeat for 3 BP readings in total.
7. Check the assessment for each reading (see "Guide for Blood Pressure Assessment" on next page).Close/exit the App.
8. To take a new BP reading for another patient, start the App again and select a new patient.

Checklist for each patient visit:

- ✓ Remember to log each patient visit and BP reading in your log sheet
- ✓ Review and assess each BP reading, advise the patient to seek care if their reading is ***High*** or ***Very High.***
- ✓ Remind patients to bring their phone and BP notebook to every visit.
- ✓ Ask patients if they are still receiving text messages.
- ✓ Remind the patient about their next visit with you.

General TIPS and Troubleshooting

- Charge your BlackBerry before going to see patients.
- Lock the BlackBerry or power off when not in use for long periods.
- Protect patient privacy and do not share the BlackBerry with others.
- Check the battery level on the BP monitor to see if you need to replace the batteries. Only use AA batteries.
- For more information, see the complete list of troubleshooting tips

GUIDE FOR BLOOD PRESSURE ASSESSMENT

Now out of date

Condition	Systolic and Diastolic	Assessment
Person under 80 years of age	<140 and < 90	**Normal**
	Systolic 140 -179 or Diastolic 90 -109	**High - Instruction:** Please advise patient to talk to their health care provider about what to do.
Person with diabetes under 80 years of age	< 130 and < 80	**Normal**
	Systolic 130 -179 or Diastolic 80 -109	**High - Instruction:** Please advise patient to talk to their health care provider about what to do.
Elderly person 80 years of age or older	<150 and <90	**Normal**
	Systolic 150 -179 and Diastolic 90 -109	**High - Instruction:** Please advise patient to talk to their health care provider about what to do.
Anyone	Systolic 180 and above or Diastolic 110 and above	**Very High - Instruction:** Please instruct patient to see their doctor or health care provider **as soon as possible.**

For any questions, please contact us at:

GENERAL TROUBLESHOOTING TIPS		
**Problem**	**Possible Reason**	**Recommended Action**
The monitor won't turn on; nothing appears on display.	Batteries are low/drained or not in correct position.	Check if batteries are in the correct position. If the problem still occurs, replace batteries with new ones.
BP cuff does not inflate.	Battery power is low	Replace all batteries with new ones.
The monitor won't take any readings.	Cuff is not positioned properly or too much arm or body movement	Adjust the cuff to fit on the patient properly and ask them to remain still and quiet during measurement. The device may also have difficulty for a weak or irregular heart beat.
The monitor is sending too many BP readings	Readings are saved on the monitor from previous patients	Write down the BP readings from the patient, check before accepting and sending readings to the server. Do NOT send /accept readings from other patients.
BP readings take a long time to show up on the BlackBerry.	You are out of range for cell coverage or the monitor is trying to find a connection	Be patient or take another reading until it shows up on the BlackBerry. Check that you are connected to the 3G network and Bluetooth connection is turned on.
BP readings take a long time to "send" to the server after I accept the reading	You are out of range for cell coverage, it is trying to find a connection	As long as you have "accepted" the readings to be sent, the readings will be transmitted to the server eventually, once a stable wireless connection is found. If you are concerned, make a note and review with the study team during a teleconference.
App says, "Connection to unknown device failed"	BlackBerry is not connected or programmed to connect to the BP monitor	Always use a BP monitor and BlackBerry that are programmed to work together. To program the devices, use 'pairing' code, Enter: **39121440.** Accept Yes to make a connection.

DREAM – GLOBAL STUDY

Who can participate in the DREAM-GLOBAL Study?

CAN Participate:

- Age 18 years or older
- Hypertension
- BP at **140/90 mmHg or higher** at the initial screening visit,
- or BP at **130/80 mmHg or higher** for patients with **diabetes**
- Hypertension for at least 12 weeks before screening and throughout the screening period
- If on BP medications, continuous drug therapy with the same dose for 8 weeks prior to and throughout the screening period
- Or no hypertension, but one or more of the following:
  - BMI > 30 kg/m2,
  - diabetes,
  - smoking,
  - previous history of coronary artery disease including stroke and heart attack.
- Written informed consent

CANNOT Participate (if any of the following):

- Change in BP medication during the 8 weeks before enrolment
- Poorly controlled hypertension with **BP at 180/110 mmHg or higher**
- No primary health care practitioner
- Unable/unwilling to visit health care provider
- Participation in a clinical trial or receipt of investigational compound or treatment in the 3 months prior to the initial screening visit.
- Unable to read the SMS text messages
- Active malignant disease (except non-melanoma skin cancer)
- Planned elective surgery during the study period except for cataract surgery
- *For BP screening study, must not be on an antihypertensive in the last 6 months.

For any questions or troubleshooting, please contact us at:

Consent Procedure Work Sheet/ Source Document

Site ID: ___ ___ ___ Pt. No. ___ __ ___

Informed Consent Process
Version: May 15, 2013

Date: ______________ Time: ____________

	Yes	No
1. Study has been fully explained to subject/family	o	o
2. Consent reviewed/discussed with subject	o	o
3. Subject/Family had opportunity to ask questions and received satisfactory answers	o	o
4. Follow up discussed	o	o
5. Subject verbalizes understanding of procedure, follow-up, risks and benefits related to the study	o	o
6. Subject denies being part of any other research study	o	o
7. Consent signed prior to any study related procedures	o	o
8. Copy of signed and dated consent given to subject	o	o

Comments:
____
____
____
____
____
____
____
____
____

Coordinator Signature: ____

DREAMGLOBAL – Cellular Safety

How to be safe with your cellular study device!

Driving

When you're on the road, safe driving is your primary responsibility. DREAM-GLOBAL is very much aware of the issue of cellular phone use and safety.

Distracted driving can be caused by a number of things including cellular phone use, eating, drinking, personal grooming, lighting cigarettes, changing radio stations, using a GPS or attending to children. To minimize distractions that may be caused by using your wireless phone in a vehicle, please keep the following information in mind:

From the Ontario Ministry of Transportation (http://www.mto.gov.on.ca/english/safety/distracted-driving/index.shtml):

Ontario's ban on hand-held devices while driving took effect on October 26, 2009.

The law makes it illegal for drivers to talk, text, type, dial or email using hand-held cell phones and other hand-held communications and entertainment devices. The law also prohibits drivers from viewing display screens unrelated to the driving task, such as laptops or DVD players, while driving. The use of hands-free devices is still permitted, and drivers may use hand-held devices to call 9-1-1.

'Hands-free' use means that apart from activating or deactivating the device, it is not held during use and the driver is not physically interacting with or manipulating it. Actions such as dialling or scrolling through contacts, or manually programming a GPS device, for example, are not allowed. If a hands-free device is used, avoid emotional or stressful conversations.

Driving requires your full attention.

Many drivers today tend to view driving, especially in familiar environments, as a simple everyday task that requires minimal attention. In fact, driving is a complex task that requires your full attention every time you get behind the wheel. At the very least you are:

- Operating a heavy piece of machinery at high speed
- Navigating across changing terrain
- Calculating speeds and distances

- Responding to other drivers, signs signals and obstacles around you

The dangers of distracted driving are real and the evidence speaks for itself: drivers who use cell phones are four times more likely to be in a collision than a driver who is focused on the road. Cell phones and other wireless communication and entertainment devices are a significant visual and cognitive distraction for drivers, with average "eyes off the road" times that increase the risk of collision considerably. At highway speed, a driver sending a simple text message travels the length of a football field without looking at the road.

Privacy

Text messaging is not considered to be secure and confidential. There is no lock function on your cell phone. This means anyone who can access your cell phone can access the information inside. If you do not want others to know about your participation in the study, we suggest that you do not share your phone with others during the study period. Messages will be sent to you about managing high blood pressure, but will not contain information about your personal health information.

In addition to not sharing your phone, please avoid giving important numbers over your wireless phone (i.e., your bank account or credit card numbers). We cannot ensure that information sent using your cellular phone will be kept confidential.
