# Supplementary material for: Analysis of the Implementation, User Perspectives, and Feedback From a Mobile Health Intervention for Individuals Living With Hypertension (DREAM-GLOBAL): Mixed Methods Study
Source: JMIR Mhealth Uhealth. 2019 Dec 9;7(12):e12639. doi: 10.2196/12639 (PMC6928701; doi:10.2196/12639)
Supplement: Multimedia Appendix 3 [file mhealth_v7i12e12639_app3.pdf]

Appendix 2 - DREAM-GLOBAL Study – SMS Bank of Text Messages

ACTIVE Type	Canada/English
Measurement normal	You are doing a good job of managing your blood pressure! Your blood pressure is **s/d** today.
Measurement high	Your blood pressure is **s/d** today, not quite at target. Please talk to your health care provider about what to do.
Measurement very high	Your blood pressure is **s/d** today – this is very high. Please see your doctor or health provider as soon as possible
Physiology/Importance of HTN Therapy	Taking your medication eases the stress on your heart and lowers your blood pressure, reducing the chance of strokes.
Risk/Benefit Side Effects	The medications provide many benefits but might have side effects. Talk to your health care provider if you have any side effects.
Drug Treatment	If you are taking your blood pressure pills according to instructions, you will get the best results.
Drug Classes for Multiple Drug Therapy	Your doctor might prescribe more than one kind of blood pressure pill for you.
Drug Classes for Multiple Drug Therapy	Most people need more than one kind of pill to control their blood pressure
Adherence	Have you taken your medication today? Your medications work much better if you take them every day at the same time
Adherence	Your blood pressure pills can be taken together.
**EVENTS Messages**	
Health Care Provider - Event	This is a reminder to see your health care provider within 14 days.
EVENTS	A friendly reminder: Have you seen your health care provider in the past 7 days? 1 = yes 0 = no
**PASSIVE Type**	**Canada/English**
Stress	Relaxation techniques like deep breathing help you control your blood pressure.
Sodium	Cooking with less salt or no salt will help to lower your blood pressure.
Sodium	For a healthier blood pressure, do your best to reduce or avoid processed meats like bacon, bologna, salami and sausage.
Sodium	Choosing foods where the label shows less than 15% of the Daily Value for sodium is one way to help lower your blood pressure.
Sodium	Choosing foods where the label shows less than 15% of the Daily Value for sodium is one way to help lower your blood pressure.
Sodium	Choosing foods that say “sodium-free”, “low sodium” or “no added sodium” will help you have a healthier blood pressure.
DASH - Fruit	Try to eat fruit more often as a snack or with meals.

Appendix 2 - DREAM-GLOBAL Study – SMS Bank of Text Messages

DASH – Vegetables	Try to increase the amount of vegetables you eat every day by having a serving at lunch and another at dinner.
DASH – Lean Meats & Poultry	When eating meat, a healthy portion is about the size of the palm of your hand.
DASH – Lean Meats & Poultry	Try meatless meals a couple of times a week, using beans or lentils, because they have less unhealthy fat.
DASH – Lean Meats & Poultry	Did you know that you can trim away the skin and fat from poultry and meat to make it healthier for your heart?
DASH – Lean Meats & Poultry	A heart healthy diet includes at least a couple of servings of fish every week, especially local fresh fish.
DASH – Lean Meats & Poultry	Healthy cooking tip: there are lots of ways to flavour food without using salt - like onions, herbs, spices, vinegar, garlic, ginger, lemons, sodium-free broth, or a bit of reduced-sodium soy sauce.
DASH – Nuts, seeds	Nuts provide fiber and minerals that are heart healthy. A healthy serving is ¼ to 1/3 cup, without added salt.
DASH – Sweets & Alcohol	It is good for your heart if you cut back on foods with lots of sugar that give you too many calories, such as pop, candy, cookies and sweets.
DASH – Sweets & Alcohol	A heart healthy diet limits alcohol to two drinks or less each day for men, and one drink or less each day for women.
DASH – Dining Out	Tip: For a healthier heart, don’t use the salt shaker.
DASH	When shopping, take a minute to read nutrition facts on the label, so you can choose foods lower in sodium, sugar and fat.
Physical Activity	Regular physical activity can lower your blood pressure. Aim for at least 30 minutes most days of the week of moderate physical activity such as walking.
Physical Activity	Walking, snowshoeing, canoeing, swimming or fitness classes all help lower blood pressure when done regularly.
Physical Activity	Don't be afraid to be active. If you have not been active for quite some time or if you are beginning a new activity or exercise program, take it gradually
Physical Activity	Regular physical activity makes your heart stronger, so it can pump more blood with less effort, lowering your blood pressure.
Physical Activity	For some people getting regular exercise is enough to reduce blood pressure.
Physical Activity	Regular exercise helps you maintain a healthy weight and also helps to control blood pressure.
Smoking	Reducing tobacco use is good for your heart and blood pressure, because the heart rate will be healthier, and the heart will get more oxygen.
Smoking	Quitting smoking is the most powerful thing you can do to improve your health.
